# Anaerobic spondylodiscitis: a retrospective analysis

**DOI:** 10.1186/s12891-022-05749-0

**Published:** 2022-08-17

**Authors:** Chien-Ting Chen, Meng-Huang Wu, Tsung-Yu Huang, Yen-Yao Li, Tsung-Jen Huang, Chien-Yin Lee, Che-Han Lin, Ching-Yu Lee

**Affiliations:** 1 Department of Orthopedic Surgery, Chang Gung Memorial Hospital, Chiayi, Taiwan; 2 Department of Orthopedics, Taipei Medical University Hospital, No.252, Wu-hsing St., Taipei, 11031 Taiwan; 3 Department of Orthopaedics, School of Medicine, College of Medicine, Taipei Medical University, No.250, Wu-hsing St., Taipei, 11031 Taiwan; 4 Division of Infectious Diseases, Department of Internal Medicine, Chang Gung Memorial Hospital, Chiayi, Taiwan; 5 International Ph.D. Program for Cell Therapy and Regeneration Medicine, College of Medicine, Taipei Medical University, No.250, Wu-hsing St., Taipei, 11031 Taiwan

**Keywords:** Anaerobic spondylodiscitis, Atypical radiographic characteristics

## Abstract

**Background:**

This retrospective study analyzed the clinical characteristics and outcomes of patients with anaerobic spondylodiscitis.

**Methods:**

From a total of 382 patients with infectious spondylodiscitis, nine patients (2.4%; two male and seven female with an average age of 67 years) with anaerobic spondylodiscitis between March 2003 and March 2017 were analyzed.

**Results:**

Most of the patients (77.8%) initially presented with afebrile back pain. Hematogenous spread occurred in seven patients and postoperative infection in two patients. *Bacteroid fragilis* was the most common pathogen isolated from three patients. Atypical radiographic characteristics, including a vertebral fracture with the preservation of disk height or coexisting spondylolytic spondylolisthesis, occurred in four patients with hematogenous anaerobic spondylodiscitis. The eradication rate of anaerobic infection was significantly higher in the patients with hematogenous infection than in those with postoperative infection (100% vs. 0%, *p* = 0.0476). Anaerobic spondylodiscitis accounted for 2.4% of cases of infectious spondylodiscitis and predominantly affected the female patients.

**Conclusions:**

Diagnostic delay may occur because of atypical spinal radiographs if the patient reports only back pain but no fever. Anaerobic infection following elective spinal instrumentation has a higher recurrence rate.

## Introduction

Pyogenic spondylodiscitis is defined as a bacterial infection involving the vertebral body and intervertebral disk and resulting from hematogenous spread or the direct inoculation of microorganisms during surgery. Pyogenic spondylodiscitis accounts for 2–7% of all musculoskeletal infections, and its incidence is 2.2–5.8 per 100,000 person-years [1]. Pyogenic spondylodiscitis is primarily caused by aerobic organisms, with *Staphylococcus aureus* being the most common causative pathogen, followed by coagulase-negative *Staphylococci*, *Escherichia coli*, and *Streptococci* [1–3]. Anaerobic spondylodiscitis is extremely uncommon and appears to account for less than 3% of pyogenic spondylodiscitis infections [4, 5]. The pathogenic organisms most frequently isolated in anaerobic spondylodiscitis are *Bacteroides*, *Propionibacterium acnes*, and *Peptococcus* [4]. Few studies have investigated anaerobic spondylodiscitis in a single institute. This study illustrates the clinical characteristics, imaging findings, and clinical outcomes of patients with anaerobic spondylodiscitis.

## Methods

### Patients

A retrospective review of patients with infectious spondylodiscitis was conducted to identify patients with anaerobic spondylodiscitis at Chang Gung Memorial Hospital between March 2003 and March 2017. This retrospective study was approved by the Ethics Committee and Institutional Review Board of Chang Gung Memorial Hospital (No. 103-2201B). Infectious spondylodiscitis was defined as a spinal infection encompassing vertebral osteomyelitis and discitis infected by anaerobic bacteria. The diagnostic impression of infectious spondylodiscitis was based on clinical presentation and imaging findings from plain radiographs and contrast-enhanced magnetic resonance imaging (MRI). When infectious spondylodiscitis was provisionally diagnosed, two sets of blood cultures were tested. Anaerobic spondylodiscitis was diagnosed when the results of the culture tests were positive for anaerobic bacteria in infected specimens obtained through computed tomography (CT)-guided biopsy or during surgery.

### Microbiological culture

All of the tissue samples were sent to the Bacteriology Laboratory of Chang Gung Memorial Hospital for microbial cultures, including aerobic, anaerobic, mycobacterial and fungal cultures. All aerobic and anaerobic plates were incubated at 37 °C. Specifically, aerobic cultures were performed using Sheep Blood Agar (BAP), Eosin Methylene Blue Agar (EMB) and Columbia Colistin-Nalidixic Agar (CAN) with 5% Sheep Blood. Aerobic plates would be kept for 7 days, during which time the results would be checked daily. Anaerobic cultures were performed using CDC Anaerobe 5% Sheep Blood Agar (CDC ANA BLD), CDC ANA BLD with phenylethyl alcohol, and Bacteroides Bile Esculin/CDC Kanamycin-Vancomycin-Laked Blood Agar (BBE/CDC KVLB). The anaerobic plates would be incubated for 14 days and the results were checked daily before discarding.

### Treatment protocol

Pyogenic spondylodiscitis is treated by administering a 3-month course of antibiotics, consisting of a minimum 2-week course of parenteral antibiotics based on the culture results and completed after the normalization of serum C-reactive protein levels and leukocyte counts. Patients who survive are followed up for a minimum of 2 years. Indications for surgical treatment include poor response to antibiotic therapy, worsening neurological impairment, substantial bony destruction with segmental instability, or postoperative infection. Surgical approaches include anterior spinal surgery (ASS), posterior spinal surgery (PSS), or combined anterior and posterior approaches. The surgical approach depends on the patient’s clinical presentation and surgeon’s preference. The ASS technique involves the sequestrectomy of the infected tissue and intervertebral body fusion with an autogenous iliac bone graft, as described in another study [6]. PSS involves laminectomy for thecal sac decompression, epidural abscess removal, transforaminal lumbar intervertebral debridement, bone graft fusion, and posterior instrumentation. CT-guided drainage is used to manage psoas muscle abscess and the acumination of purulent fluid in the vertebral body or disk space with or without anterior epidural abscess when surgical treatment is not performed.

### Data assessment

Patient characteristics, underlying diseases, laboratory data at the time of presentation, bacterial culture findings, and outcomes were reviewed from the electronic database at our hospital. Recurrent infection was defined as relapsed anaerobic spinal infection within 1 year of infection resolution at the time of hospital discharge. Deaths were recorded according to whether the death was related to anaerobic spondylodiscitis.

**Fig. 1:**
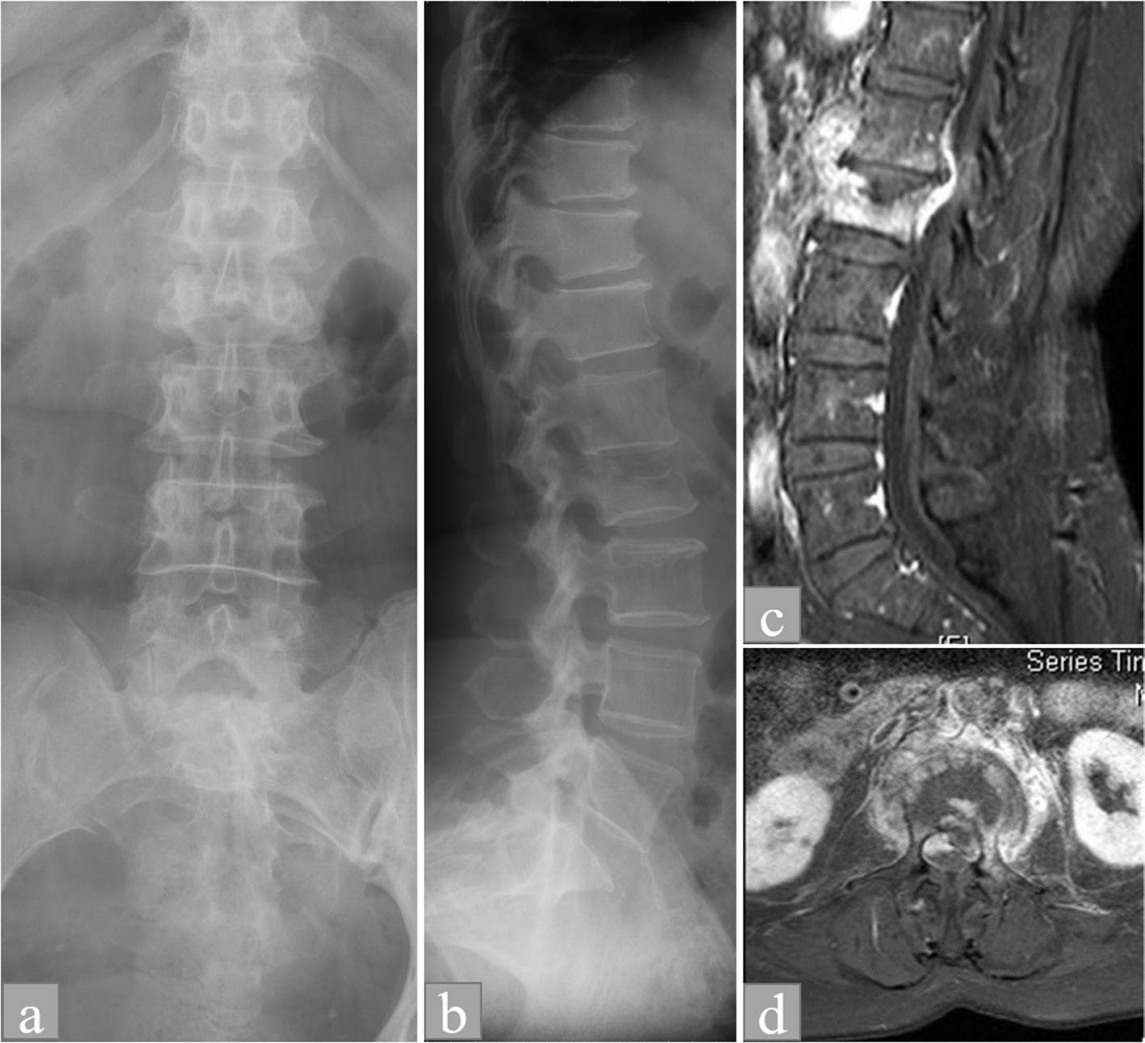
Anaerobic spondylodiscitis mimicking a fragile vertebral body compression fracture in a 72-year-old female (No. 3) with chronic lower back pain in the absence of fever. **A** Loss of vertebral body height without the widening of the interpedicular distance and interspinal process distance was observed at the L2 vertebra in the anteroposterior view of the lumbar spine radiograph. **B** Compression fracture of the L2 vertebra with preserved disk height was observed as an anterior wedging deformity and vertebral body height loss in the lateral and anteroposterior view of the lumbar spinal radiograph. **C** Heterogeneous enhancement of the L2 vertebral body and prevertebral space was observed on a sagittal gadolinium-enhanced fat-suppressed T1-weighted magnetic resonance image. **D** Enhancement of the paraspinal soft tissue muscle surrounding the L2 vertebral body. A spinal epidural abscess was observed as an epidural mass with central hypointensity and surrounding linear enhancement on an axial view of contrast-enhanced T1-weighted imaging

**Fig. 2:**
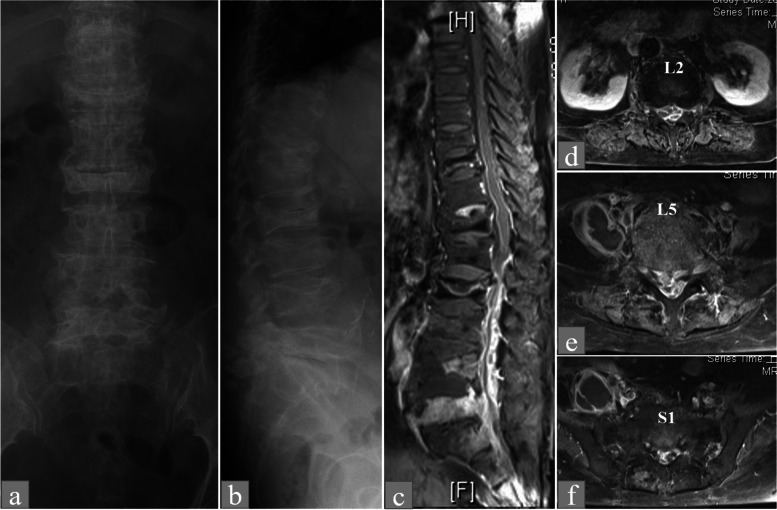
71-year-old female (No.7) with anaerobic spondylodiscitis presenting with multiple vertebral body compression fractures. **A** Lumbar anteroposterior view displaying multiple vertebral body compression fractures. **B** Intervertebral cleft within an L2 vertebral body compression fracture in the lumbar lateral and anteroposterior view. **C** Sagittal gadolinium-enhanced fat-suppressed T1-weighted imaging of abscess formation within the L2 vertebral body, and osteomyelitis in the L4 and L5 vertebral bodies with purulent collection in the intervertebral space (**D**, **E**, **F**) Contrast-enhanced T1-weighted axial imaging of a circumferential spinal epidural abscess observed at L2–S1 levels and a right-side iliopsoas muscle abscess

**Fig. 3:**
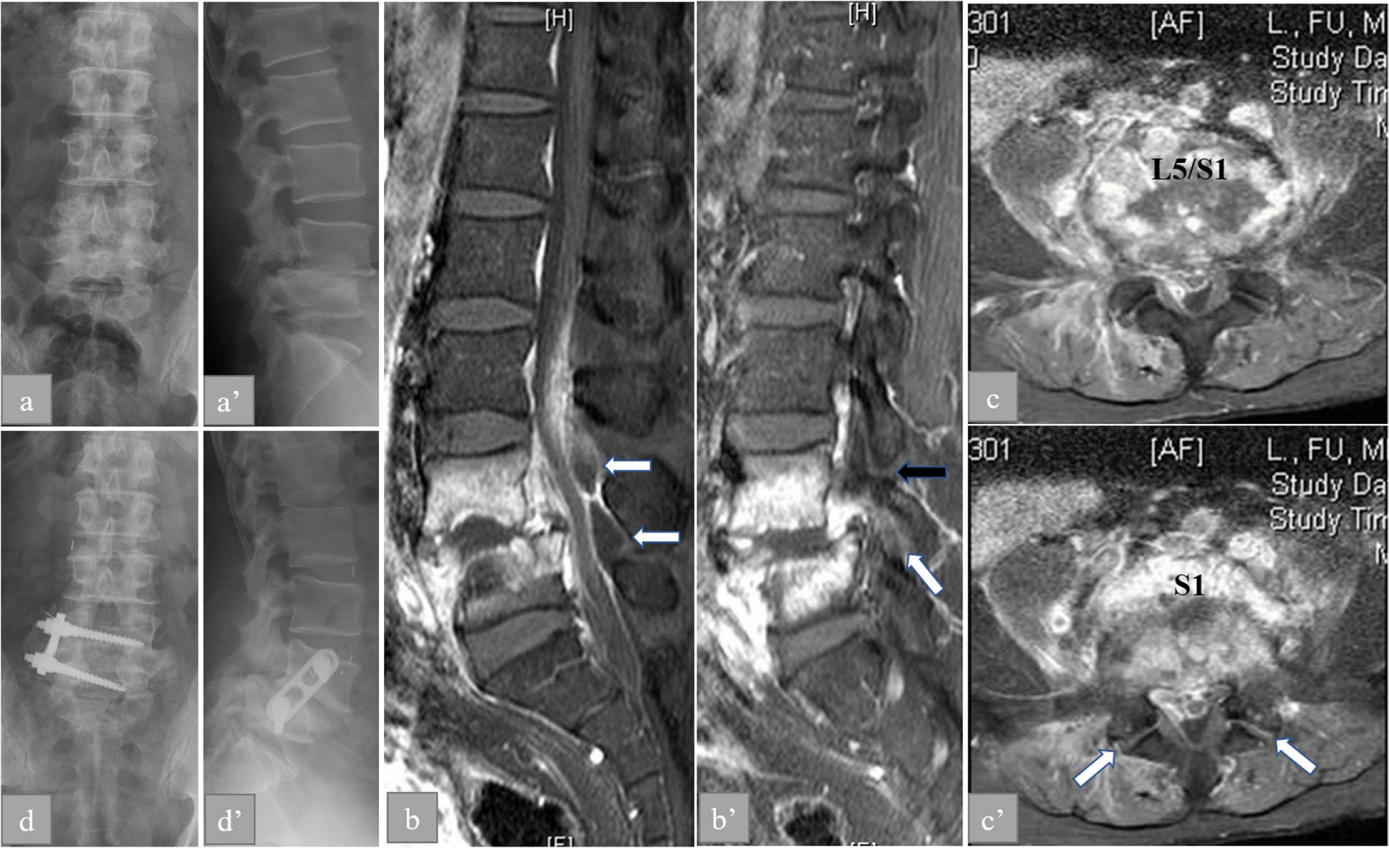
Anaerobic spondylodiscitis with concomitant spondylolytic spondylolisthesis in a 53-year-old male (No. 1). **A**1, 2 Grade 2 spondylolisthesis with pars interarticularis deficiency at L5–S1 observed on plain radiographs. **B**1 Infectious spondylodiscitis at L5–S1 with a destroyed disk on sagittal enhanced T1-weighted magnetic resonance imaging (MRI). The dorsal epidural abscesses are visible (white arrows). **B**2 Heterogeneous enhancement of the lumbosacral facet joint indicating that the facet joint was destroyed through infection (white arrow). Collection of purulent pus extended to the pars interarticularis deficiency (black arrow). **C**1, 2 Heterogenous enhancement of L5–S1, prevertebral space, bilateral foramina, and spinal canal. The collection of pus was acuminated in the lumbosacral facet joint space (white arrow). **D**1, 2 Anaerobic spondylodiscitis with concomitant spondylolytic spondylolisthesis diagnosed through contrast-enhanced MRI and bacterial cultures. The patient underwent anterior sequestration and reconstruction with interbody fusion with autogenous iliac crest and instrumentation. The solid bony fusion without the loosening of the instrumentation was observed at the 2-year follow-up

Imaging parameters recorded were atypical radiographic characteristics, psoas muscle abscess, and epidural abscess. Typical radiographic features included a reduction in disk height caused by the erosion and destruction of adjacent vertebral endplates [7]. Atypical radiographic characteristics were defined as the destruction of a vertebral body with sparing of the intervertebral disk space mimicking a vertebral compression fracture (Figs. 1 and 2) or coexisting spondylolytic spondylolisthesis (Fig. 3) [8, 9]. Spinal epidural abscess was diagnosed through MRI as an epidural mass with iso-intensity or hypo-intensity on T1-weighted images, hyperintensity on T2-weighted images, and the linear enhancement of nonenhancing purulent matter [10].

### Statistical analyses

Statistical analyses were performed using SPSS 12.0 software for Windows (SPSS Inc., Chicago, IL, USA). Fisher’s exact test was used for dichotomous variables, and statistical significance was set at a *p* value of < 0.05. Descriptive data are presented as the mean with standard deviation for quantitative variables and as the number with frequency for categorical variables.

## Results

### Patient characteristics

A total of 382 patients with infectious spondylodiscitis were identified between March 2003 and March 2017. Nine patients (2.4%; two male and seven female), with an average age of 67 years (range, 53–79 years), received a diagnosis of anaerobic spondylodiscitis during this period. The average follow-up period was 2.7 years (range, 2–5 years) with the exception of two patients who died during treatment. The clinical data are presented in Tables 1 and 2. All the patients had at least one immunocompromised disease. The most common presenting symptom was chronic back pain for more than 1 month. Fever occurred in two of the patients (22.2%), and the lumbar spine was affected in all patients. Single-level spinal infection occurred in six patients (66.7%), and two or more levels were involved in three patients (33.3%). Anaerobic bacteremia occurred in five patients (55.6%) with the same pathogen as that identified in the spinal lesion culture obtained from those five patients. Regarding the route of infection, hematogenous spread occurred in seven patients (77.8%) and direct inoculation during surgery in two patients (22.2%). In addition to antibiotic therapy, three patients received CT-guided drainage and six patients underwent surgical therapy. In the hematogenous-infection group, five patients treated through CT-guided drainage or surgical intervention were cured without infection recurrence and two patients died for reasons not related to anaerobic spondylodiscitis. One of these patients (number 6) died of pneumonia caused by multidrug-resistant *Klebsiella pneumonia*, which occurred 2 months after receiving a complete treatment course for spinal infection and being discharged from the hospital. The other patient (number 7) died of Stevens–Johnson syndrome caused by proton pump inhibitors in the fourth week of hospitalization. In the postoperative-infection group, both patients developed recurrent infection.

**Table 1:** Patient characteristics

No.	Sex	Age	Comorbidity	Symptoms at first visit	Onset (month)	Characteristics of plain radiographs	Infection level
1	male	53	DM	back pain, sciatica	4	spondylolisthesis	L4,5
2	male	68	HCC, HBV	fever, back pain	1	typical features	L3,4
3	female	72	DM	back pain	1	compression fracture	L2
4	female	57	DM	back pain, sciatica	1	typical features	L5, S1
5	female	64	CKD	fever, back pain	1	typical features	L3,4
6	female	79	DM, LC	back pain, sciatica	4	compression fracture	T12, L4,5
7	female	71	CKD	back pain, sciatica, weakness	3	compression fracture	L2,4,5
8	female	63	DM	back pain, sciatica	3	typical features	L2,3
9	female	73	CKD	back pain, weakness	3	typical features	L2,3,4,5

Abbreviation: *No.* number, *DM* diabetes mellitus, *HCC* hepatocellular carcinoma, *HBV* hepatitis B virus, *CKD* chronic renal disease, *LC* liver cirrhosis, *BP* back pain, *S* sciatica, *F* fever, *W* weakness

Typical features: a reduction in disk height caused by the erosion and destruction of adjacent vertebral endplates

**Table 2:** Patient characteristics

No.	Route of infection	Microbe	Bacteremia	Intervention therapy	Antibiotics (duration, week)	Outcome
1	Hematogenous	*Eikenella corrodens*	No	ASS	IV amoxicillin (2) PO amoxicillin (10)	Cured
2	Hematogenous	*Prevotella* sp.	Yes	CT-guided drainage	IV metronidazole (4) PO Penicillin-V (8)	Cured
3	Hematogenous	*Peptostreptococcus* sp.	No	CT-guided drainage	IV Penicillin-G (4) PO Penicillin-V (8)	Cured
4	Hematogenous	*Fusobacterium* sp.	No	PSS	IV metronidazole (3) PO metronidazole (9)	Cured
5	Hematogenous	*Prevotella* sp.	Yes	PSS	IV moxifloxacin (3) PO moxifloxacin (9)	Cured
6	Hematogenous	*Bacteroid fragilis*	Yes	CT-guided drainage	IV metronidazole (4) PO metronidazole (8)	UD
7	Hematogenous	*Bacteroid fragilis*	Yes	ASS + PSS	IV metronidazole (3)	UD
8	Postoperative (11 month)	*Peptostrepto*. *magnus*, *Escherichia coli*, *Enterococcus faecalis*	No	ASS + RPSI	IV clindamyclin plus IV ampicillin (4) PO clindamyclin plus PO ampicillin (8)	Recurrence
9	Postoperative (7 month)	*Bacteroid fragilis*	Yes	PSS	IV metronidazole (3) PO metronidazole (9)	Recurrence

Abbreviation: *No.* number, *Peptostrepto. Peptostreptococcus*, *EA* epidural abscess, *PA* psoas muscle abscess, *ASS* anterior spinal surgery, *PSS* posterior spinal surgery, *CT* computed tomography, *RPSI* removal of posterior spinal instrumentation, *IV* intravenous, *PO* oral, *UD* unrelated death

### Imaging presentation

Imaging findings are presented in Table 3. In radiographic findings, atypical characteristics were identified in four (57%) of the seven patients with hematogenous anaerobic spondylodiscitis: vertebral compression fractures in three patients and pathologic spondylolisthesis in one patient. In MRI findings, epidural abscess was identified in eight patients (89%) and psoas abscess in seven patients (78%).

**Table 3:** Imaging findings of patients with anaerobic spondylodiscitis

Imaging findings	Hematogenous infection (*n* = 7)	Postoperative infection (*n* = 2)	Total patients (*n* = 9)
Atypical characteristics on plain radiographs	4 (57)	0	4 (44)
Epidural abscess on MRI	7 (100)	1 (50)	8 (89)
Psoas muscle abscess on MRI	6 (86)	1 (50)	7 (78)

Data: number (%)

Atypical characteristics: a vertebral body compression fracture with preservation of disk height or coexisting spondylolytic spondylolisthesis

*MRI* magnetic resonance imaging

### Microbiological findings

The microbiological findings are presented in Table 4. In the hematogenous-infection group, *Bacteroides fragilis* and *Prevotella were isolated from two patients,* and *Peptostreptococcus*, *Eikenella corrodens*, and *Fusobacterium* were isolated from one patient. In the postoperative-infection group, *B. fragilis* and polymicrobial bacteria (*Peptostreptococcus magnus*, *E. coli*, and *Enterococcus faecalis*) were isolated from one patient.

**Table 4:** Microbiological findings

Anaerobic microbes	Hematogenous infection (*n* = 7)	Postoperative infection (*n* = 2)	Total patients (*n* = 9)
*Bacteroid fragilis*	2 (29)	1 (50)	3 (33)
*Peptostreptococcus* sp.	1 (14)	1 (50)	2 (22)
*Prevotella* sp.	2 (14)	0	2 (22)
*Eikenella corrodens*	1 (14)	0	1 (11)
*Fusobacterium* sp.	1 (14)	0	1 (11)

Data: number (%) or number

### Outcome analysis

The eradication rate of anaerobic infection was significantly higher in the patients with hematogenous infection than in those with postoperative infection (100% vs. 0%, *p* = 0.0476) (Table 5). The hematogenous-infection group experienced no recurrence of infection. By contrast, in the postoperative-infection group, two patients experienced recurrence excluding the two deaths from nonspinal infections. Of the two patients with recurrence, one (number 8) had a surgical wound on the back that had failed to heal 3 weeks after hospital discharge and received repeated surgical debridement to cure the wound infection and the other (number 9) had recurrent infection 2 weeks after hospital discharge and had no recurrence following antibiotic therapy alone.

**Table 5:** Eradication of infection caused by hematogenous spread or postoperative infection

	Hematogenous spread *n* = 7	Postoperative infection *n* = 2	*P* value
Eradication of infection	5 (100)	0	0.0476^*^
Recurrent infection	0	2 (100)	

Fisher’s exact test was used for the statistical analysis. Data: number (%). * difference is significant (*p* < 0.05)

## Discussion

This is the first case series to discuss the clinical presentation of anaerobic spondylodiscitis in a single tertiary-care hospital (Chiayi Chang Gung Memorial Hospital). The prevalence of anaerobic spondylodiscitis in this study was 2.4% among the 382 patients with infectious spondylodiscitis. All the patients in this study had at least one immunocompromised disease, and more than half had diabetes mellitus. The main symptom of anaerobic spondylodiscitis was chronic back pain in the absence of fever. To date, only one large-scale case series has been conducted in patients (*n* = 29) with anaerobic spondylodiscitis caused by *Propionibacterium acnes* [11]. Consistent with our findings, all patients presented with back pain and most of them were afebrile*.* By contrast, fever was present in up to 60% of cases of pyogenic aerobic spondylodiscitis and is a medical triad indication (back pain, fever, and neurological deficit). Insidious symptoms, such as chronic back pain and being afebrile, may result from low anaerobic bacterial virulence and a slow growth rate. Furthermore, in a literature review, aging [12, 13], nonhematologic malignancy [13], chronic renal insufficiency [14], and diabetes mellitus [15] were risk factors for afebrile bacteremia. Therefore, anaerobic spondylodiscitis should not be excluded because of a lack of fever, especially in patients with comorbidities such as liver cirrhosis, chronic renal insufficiency, and diabetes mellitus.

Pyogenic spondylodiscitis has been demonstrated to have a male predominance, with the male to female ratio is as high as 3:1 [2]. By contrast, this study revealed that female patients predominated (77.8%) among those with anaerobic spondylodiscitis. Anaerobic species inhabit the mucosal surfaces in healthy individuals including the oral cavity and the gastrointestinal, urinary, and female genital tracts [16] Microbial colonization of the female genital tract indicates that anaerobic bacteria outnumber aerobic bacteria in a ratio of 10:1 [17]. In addition, menopause contributes to the epithelial atrophy of the genital mucosa and alters the pH level of the vaginal environment, both of which disturb the antimicrobial activity of the female genital tract, replacing healthy microflora with invading anaerobic bacteria, such as *B. fragilis*, *Prevotella*, *Fusobacterium*, and *Peptostreptococcus* [18–20]. Similarly, the most common pathogens in this study were *B. fragilis* and *Peptostreptococcus*, followed by *Prevotella*. Therefore, unlike aerobic spondylodiscitis, anaerobic spondylodiscitis was predominant in the female patients.

The plain radiographic features of bacterial spondylodiscitis mostly exhibit a reduction in disk height and irregularities in both adjacent endplates, and atypical characteristics on plain films include single incontiguous vertebral destruction with the preservation of disk height, mimicking vertebral compression fractures, which often occur in spinal tuberculosis [8, 21]. Pathologic spondylolisthesis secondary to infection or infectious spondylodiscitis with concomitant spondylolisthesis can be considered an atypical radiographic characteristic of infectious spondylodiscitis but with extremely rare occurrence [9]. In this study, atypical radiographic characteristics accounted for more than half (57%) of the patients with hematogenous anaerobic spondylodiscitis. Few studies have investigated the radiographic features of anaerobic spondylodiscitis. Similar to our findings, Dewan et al. reported that *Actinomyces* affected multiple vertebral bodies, only sparing the intervertebral disks, and Pilmis et al. reported the case of a patient with spondylodiscitis caused by *Parvimonas micra* who presented with vertebral compression fracture [22, 23]. In a literature review, spinal infection with spondylolisthesis occurred mainly in spinal tuberculosis [9, 24–26]. Most case reports indicated that spinal tuberculosis coexists with spondylolisthesis [9, 27, 28], and rare cases suggested that tuberculosis predated the development of spondylolisthesis [25]. In patient 1 of this study, pathologic spondylolisthesis secondary to infection could not be identified because a lumbar spine X-ray was not performed prior to spinal infection. However, Suppurative erosions of the L5 pars interarticularis and the lumbosacral facet joints were observed as well as destruction of the L5-S1 intervertebral disc, which may lead to spondylolisthesis. It is believed that when the vertebrae (for example, an intervertebral disc, facet joints, or the pars interarticularis) are extensively destroyed by infection, the stress damage to the neural architecture may lead to spondylolisthesis whether or not there is evidence of non-slippage or non-defect of the isthmus before infection [9, 26].

The reason for the appearance of atypical characteristics on plain films is unclear. First, we explored the spinal vascular system to explain the phenomenon of vertebral destruction with the preservation of disk height. *S. aureus*, the most common aerobic pathomicroorganism, spreads to the spine through the arteriolar network, which originates in segmental arteries and has end vessels at the superior and inferior endplates of each vertebral body [29]. By contrast, anaerobic bacteria, which mostly inhabit the mucosal membrane surface of the gastrointestinal tract and female genital tract, usually spread from the pelvic veins to the center of vertebral bodies through the paravertebral venous plexus or the Batson venous plexus, whereas aerobic bacteria tend to spread to the spine through the arteriolar network [30, 31].

The intervertebral disc (IVD) degeneration caused by low-grade bacterial infection is a research hotspot. In a study conducted by Ozger in 2020, which included 33 patients with single-level lumbar disc herniation (LDH) undergoing microdiscectomy and analyzed the frequency of aerobic bacterial infection in disc tissue, the prevalence of subclinical aerobic bacterial infection, which was caused by coagulase-negative *staphylococci* and *Enterobacteriaceae*, was found to be 12.12% in patients with LDH [32]. In addition, more studies have found that low-virulence anaerobic bacteria (LVAB) were the major pathogen in the culture-positive discs from discectomy in the treatment of intravertebral degenerative disc diseases [33, 34]. A systematic review and meta-analysis study of bacterial cultures from 2084 discectomies (including 16 articles) showed that bacteria were present in 25.3% of discs and *Propionibacterium acnes* accounted for 56.4% of bacteria-positive discs [35]. The association of the bacterial presence with Modic changes remains controversial. Tang and colleagues found that the presence of bacteria was significantly associated with Modic changes [36], but the meta-analysis study was unable to demonstrate the positive association [35]. One study reported that there was no post-operative surgical wound infection in patients with the presence of bacterial growth in their disc culture [32]. The mechanism by which the hypo-virulent bacteria reside in IVD remains unclear. There is currently no evidence that the presence of LVAB in IVD is associated with the development of anaerobic spondylodiscitis. Further research is needed to investigate the exact mechanism.

Spinal epidural abscess occurred in eight patients (88.9%) in our study. However, reports on the incidence of epidural abscess in patients with anaerobic spondylodiscitis are lacking. Approximately 10.4–28.1% of patients with pyogenic aerobic spondylodiscitis develop epidural abscess, and a relatively high incidence of epidural abscess (44%) was noted in patients with multidrug-resistant bacterial spondylodiscitis [37, 38]. We revealed that the patients with anaerobic spondylodiscitis had a high incidence of epidural abscess, similar to that of multidrug-resistant bacterial spondylodiscitis, indicating that anaerobic bacteria are highly virulent.

Most guidelines recommend 6–12 weeks of antibiotic treatment for infectious spondylodiscitis [39, 40]. CT-guided drainage can be employed for psoas muscle abscess or paraspinal abscess [41]. Surgical indications for infectious spondylodiscitis include poor response to antibiotic therapy, impaired neurologic deficits, and significant bony destruction with segmental instability [42]. Regardless of the surgical approaches used, the purpose of surgical interventions is to eradicate infectious tissue, drain abscesses, decompress nerves, and stabilize spinal segments through the restoration of spinal alignment. When patients do not yet have surgical indications, CT-guided drainage appears to be the primary treatment for purulent pus in the intervertebral and epidural space [43–45]. In general, antibiotic therapy alone has a higher rate of treatment failure in implant-associated spinal infections because the bacterial biofilm on the implant surface can resist antimicrobial drugs. Hence, aggressive surgery combined with antibiotic therapy is the mainstay of treatment for postoperative instrumented spinal infection [46, 47].

In this study, in all the patients with hematogenous anaerobic spondylodiscitis who survived, the infection was successfully eradicated without recurrence after a complete course of treatment, whether through CT-guided drainage or surgical treatment. By contrast, both the patients with postoperative anaerobic infection experienced recurrent infection after surgical debridement and removal of the implant and required a second therapy to be cured. The relapse rate of infection was significantly higher in the patients with postoperative anaerobic infection than in those with hematogenous infection. Similarly, in a retrospective study comparing the clinical outcomes of patients with pyogenic postoperative and native vertebral osteomyelitis, the treatment failure and relapse rates at 12 months were higher in patients with postoperative vertebral osteomyelitis [48]. The management of postoperative instrumented spinal infection may be more challenging than that of hematogenous spinal infection, especially in the case of delayed infection [49]. Both postoperative spinal infections in our study involved delayed infection more than 7 months after spinal instrumentation. Most studies have demonstrated that anaerobic infection is more likely to cause delayed infection following spinal instrumentation. The latency period between the inoculation of microorganisms and the presentation of symptoms is 4–5 months but can be as long as 4 years [50–52]. Delayed infection following instrumentation is caused by anaerobic microorganisms producing biofilms [53, 54] which make it difficult to eradicate the infection [49]. Therefore, on the basis of these findings, the authors suggest that the successful treatment of instrumented spinal anaerobic infection requires prolonged antibiotic therapy and more aggressive surgical debridement.

The study has some limitations. First, this is a case series including a small number of patients, which might contribute to the rarity of anaerobic spine infection. Second, the surgical treatment was heterogeneous. The surgical approaches and methods depended on the location and extent of the lesion, presence of psoas muscle or epidural abscess, degree of neurologic deficit, involvement of vertebral fractures, and degree of spinal deformity as well as the surgeon’s preference.

## Conclusion

The incidence of anaerobic spondylodiscitis accounts for 2.4% of infectious spondylodiscitis. Female sex and immunocompromised diseases are both risk factors for anaerobic spondylodiscitis. The patients often present with insidious backache and a lack of fever, and atypical radiographic characteristics may develop in hematogenous anaerobic spondylodiscitis. Diagnostic delay may occur because of atypical spinal radiographs if the patient reports only back pain but no fever; hence, early diagnosis is possible with the aid of advanced imaging and microbial cultures. Delayed-onset anaerobic infection mostly occurs after elective spinal instrumentation and has a higher recurrence rate. Prolonged antibiotic therapy and more aggressive surgical debridement are required to eradicate the infection in delayed anaerobic infection following spinal instrumentation.

## Data Availability

The data used to support the findings of this study are available from the corresponding author upon request.

## Abbreviations

MRI: Magnetic resonance imaging
CT: Computed tomography
ASS: Anterior spinal surgery
PSS: Posterior spinal surgery
BAP: Sheep Blood Agar
EMB: Eosin Methylene Blue Agar
CAN: Colistin-Nalidixic Agar
CDC ANA BLD: CDC Anaerobe 5% Sheep Blood Agar
BBE/CDC KVLB: Bacteroides Bile Esculin/CDC Kanamycin-Vancomycin-Laked Blood Agar
